# Design of the MEMS Piezoresistive Electronic Heart Sound Sensor

**DOI:** 10.3390/s16111728

**Published:** 2016-11-07

**Authors:** Guojun Zhang, Mengran Liu, Nan Guo, Wendong Zhang

**Affiliations:** 1 Science and Technology on Electronic Test & Measurement Laboratory, North University of China, Taiyuan 030051, China; liumengran1991@163.com (M.L.); guonan0902@163.com (N.G.); zhangwendongnuc@163.com (W.Z.); 2 Key Laboratory of Instrumentation Science & Dynamic Measurement, Ministry of Education, North University of China, Taiyuan 030051, China

**Keywords:** MEMS, heart sound sensor, stress concentration, signal-to-noise ratio

## Abstract

This paper proposes the electronic heart sound sensor, based on the piezoresistive principle and MEMS (Micro-Electro-Mechanical System) technology. Firstly, according to the characteristics of heart sound detection, the double-beam-block microstructure has been proposed, and the theoretical analysis and finite element method (FEM) simulation have been carried out. Combined with the natural frequency response of the heart sound (20~600 Hz), its structure sizes have been determined. Secondly, the processing technology of the microstructure with the stress concentration grooves has been developed. The material and sizes of the package have been determined by the three-layer medium transmission principle. Lastly, the MEMS piezoresistive electronic heart sound sensor has been tested compared with the 3200-type electronic stethoscope from 3M (São Paulo, MN, USA). The test results show that the heart sound waveform tested by the MEMS electronic heart sound sensor are almost the same as that tested by the 3200-type electronic stethoscope. Moreover, its signal-to-noise ratio is significantly higher. Compared with the traditional stethoscope, the MEMS heart sound sensor can provide the first and second heart sounds containing more abundant information about the lesion. Compared with the 3200-type electronic stethoscope from 3M, it has better performance and lower cost.

## 1. Introduction

Auscultation is an important routine examination method in clinical diagnosis. Before the nineteenth century doctors could only put the ear on the patient’s chest for “direct auscultation”. In 1816, French doctor Laennec invented the stethoscope; therefore, “indirect auscultation” becomes possible and the discipline of cardiac auscultation is formed, which has greatly promoted the development of medicine [1]. However, the traditional stethoscope has poor low-frequency response, and diagnosis highly depends on the subjective judgment of doctors. In addition, the human ear is limited by the inherent limitation whose most sensitive frequency range is 1000~3000 Hz, which is with poor sensitivity for low-frequency sounds [2]. However the clinically valuable heart sound frequency range is often concentrated in the range of 20~600 Hz, so some important heart sounds with low frequency and small intensity are difficult to capture [3,4].

In 1999, the American 3M Littmann Company (São Paulo, MN, USA) developed and produced an electronic stethoscope, which was the first to apply electronic technology to auscultation [5]. The traditional stethoscope has been replaced by the heart sound electronic auscultation system, which has the advantages of high accuracy, real-time waveform display, ease of use, low cost, and small volume. The common sound sensor used by electronic stethoscopes is the microphone [6]. The microphone is divided into electret, coil, and capacitance, and electret is the most commonly used. The electronic stethoscope converts the heart sound signals to analog signals, which are amplified, and then the analog signals are converted to digital signals for further processing and transmission. The microphone-type electronic stethoscope receives the heart sound signals via the microphone in the cavity, which is mounted behind the diaphragm. The vibration of the diaphragm causes sound pressure in the cavity, the microphone receives the sound pressure and converts it to an electrical signal. Since the sound propagates through two layers of diaphragm and the internal cavity, namely a three-layer medium, the energy transfer is limited and the sensitivity of the stethoscope is low.

MEMS technology has been applied to the detection, diagnosis, and therapy, which plays an important role in the improvement of medical devices and the prevention, diagnosis and treatment of diseases [7,8]. The wireless endoscope technologies of M2A developed by Israeli Given Imaging Company (Tel Aviv-Yafo, Israeli) and NORIKA3 developed by Japanese RF Company (Nagano-ken, Japan) have been applied to clinical practice [9,10,11]. Thousands of biomolecules can be integrated in a centimeter-scale chip by MEMS technology, which could be made into the bio-chip. The bio-chip can show different allergic reactions for the various biochemical reactions to realize the test and analysis of the genes, antigens, ligands and other bioactive substances. MEMS technology can be also used in genetic analysis and genetic diagnosis [12,13,14]. Therefore, MEMS piezoresistive pressure sensors, with the advantages of good performance at low frequencies, comprised of mature and controllable MEMS piezoresistive technology, with low cost, linear output, and simple circuitry, have been widely used in medical diagnostic systems [15].

This paper proposes an electronic heart sound sensor with a high signal-to-noise ratio and real-time waveform display based on MEMS technology and the piezoresistive principle. Combined with further clinical experience, preliminary diagnosis can be performed by the electronic heart sound sensor and the accuracy of heart attack diagnosis can be improved.

## 2. Modelization

### 2.1. Microstructure Design

**Figure 1:**
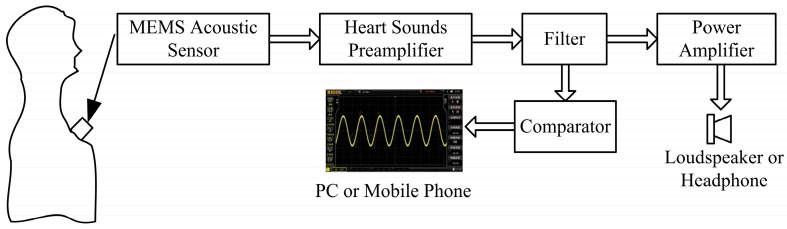
Overall system of the MEMS electronic heart sound sensor.

The design of the overall system is shown in Figure 1. The auscultation probe of the MEMS electronic heart sound sensor is affixed to the registry and receives the faint heart sound signals. The heart sounds are transferred to the filter after the pre-amplifier to filter the high-frequency noise of the filter and environment and the high frequency components of the signals. Then the signals are transferred to a PC or mobile phone for real-time display after the comparator. The signals also would be transmitted to a loudspeaker or headphones for listening after the power amplifier [16].

A piezoresistor has been chosen as the sensitive element of the MEMS electronic heart sound sensor. Based on the working principle of the piezoresistor sensor, when the piezoresistor is subjected to tensile stress or compressive stress, the migration rate changes and the resistivity changes, and then the value of the piezoresistor changes. Piezoresistors have been implanted into the stress-sensitive location, connecting into a Wheatstone bridge. The Wheatstone bridge can detect the changes of the piezoresistors so that the forces suffered by the microstructure can be obtained. Therefore, the heart sound signal can be detected by the MEMS electronic heart sound sensor.

**Figure 2:**
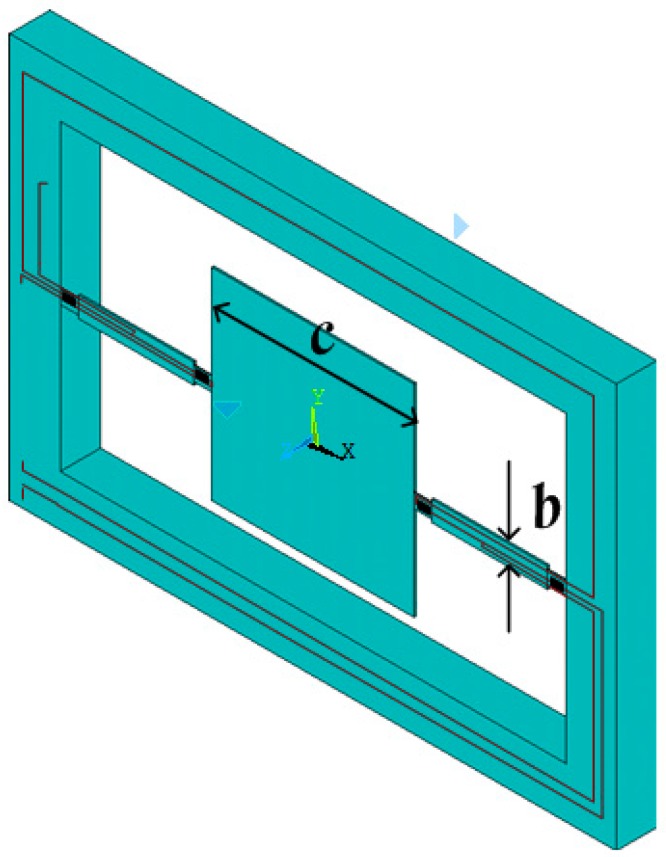
Sensitive microstructure of the MEMS electronic heart sound sensor.

**Figure 3:**
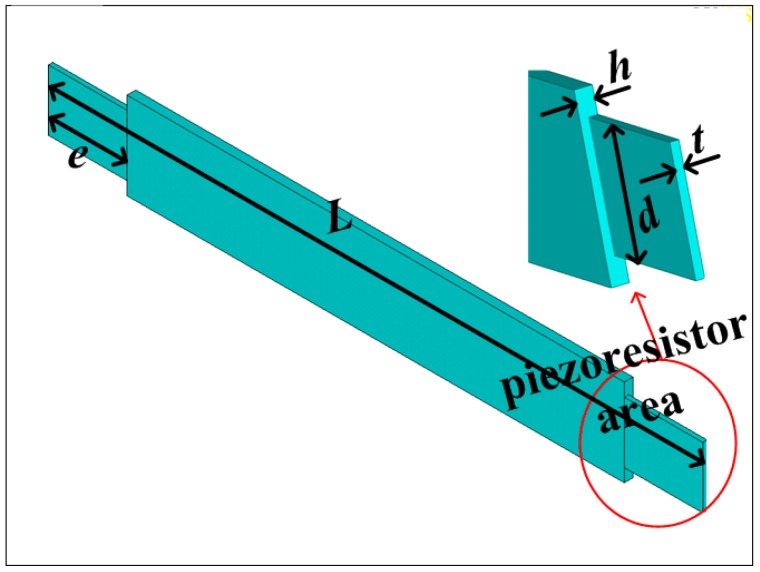
Schematic of the cantilever beam.

The MEMS electronic heart sound sensor is only used to detect the vibration strength information of the heart sound signal, and the noise interference in the other direction needs to be suppressed. Thus, this paper proposes the double-beam-block sensitive microstructure of a MEMS electronic heart sound sensor. It should maximally pick up the heart sound vibration signal and achieve the best performance under the heart sound frequency range condition. However, the sensitivity and the first-order resonant frequency of the MEMS electronic heart sound sensor restrain each other [17]. To solve this problem, a scheme of stress concentration has been proposed. The frequency band should meet the design requirements and, at the same time, the grooves should be placed on the beam near the frame and close to the mass block to concentrate the stresses into the grooves. The piezoresistors are implanted on grooves to improve the change rate of the piezoresistors and develop the ability of detecting the faint heart sounds. The sensitive microstructure of the MEMS electronic heart sound sensor is shown in Figure 2 and a schematic of the cantilever beam is shown in Figure 3. Where *L*, *b*, and *h* are respectively the length, width and thickness of cantilever beam; *c* and *h* are, respectively, the side length and thickness of mass block; *e*, *d*, and *t* are, respectively, the length, width, and thickness of the cantilever beam in the piezoresistor area.

**Figure 4:**
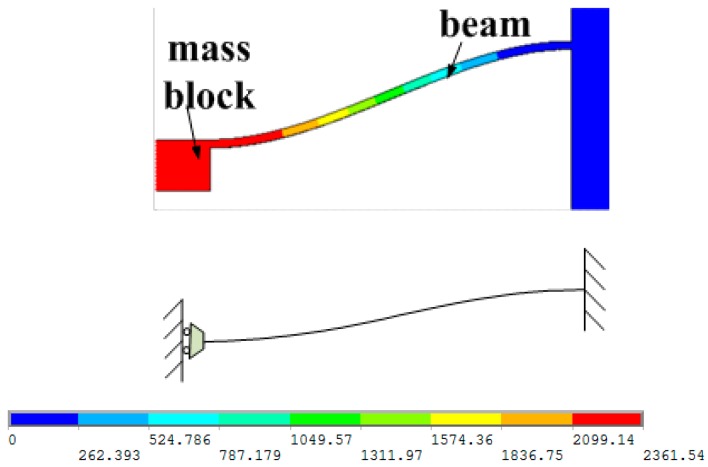
Microstructure mechanics model.

When the MEMS electronic heart sound sensor is at work, the mass block of the microstructure can be approximately seen as a rigid body, which is mainly used to detect heart sound signals. The detection beams can be considered as the elastic support beams, as shown in Figure 4. The colors represent the displacement of the microstructure.

The stress on the beam can be expressed as Equation (1), where, 0 ≤ *x* ≤ *L*:

(1) $$\sigma (x)=\frac{6b}{Lh^{2}}(\frac{7}{18}Lm_{1}+\frac{2}{3}m_{2}+\frac{1}{18}cm_{2}^{2}-m_{1}x+\frac{m_{1}x^{2}}{2L})F$$

The resonant frequency of the microstructure can be expressed as Equation (2):

(2) $$f=\frac{1}{2\pi }\sqrt{\frac{Ebh^{3}}{12m_{2}L^{3}}}\sqrt{\frac{6L^{2}+12L+8}{2L^{4}+7L^{3}+10.5L^{2}+8L+8/3}}$$

where, *m*_1_ is the mass of the cantilever beam; *m*_2_ is the mass of the mass block; *E* is the Young’s modulus of the silicon material.

Considering process limitations, performance indicators, and volume, the size parameters of the MEMS electronic heart sound sensor microstructure are determined by iterative analysis, as shown in Table 1. 

### 2.2. Simulation

**Figure 5:**
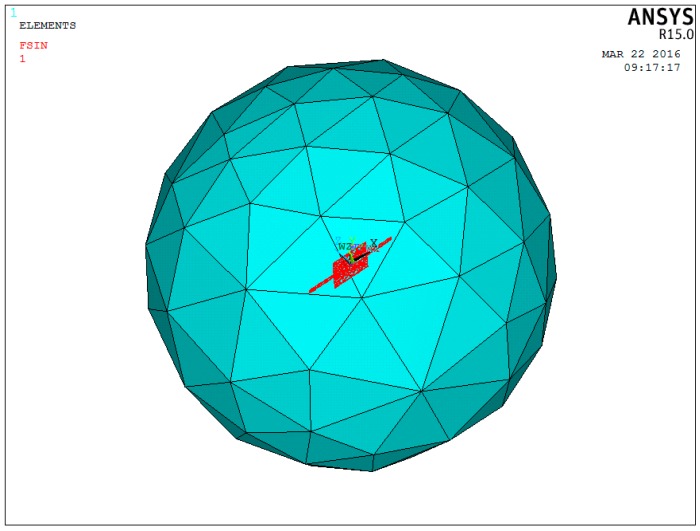
Coupling model.

**Figure 6:**
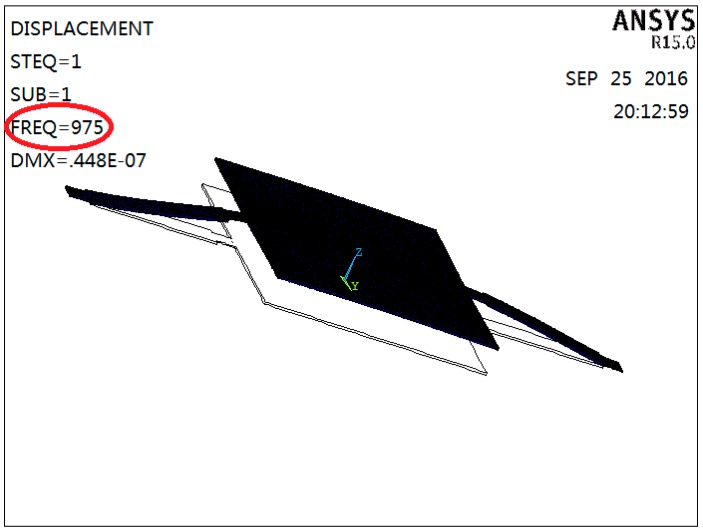
Coupling modal analysis results.

The material of the microstructure is silicon, which has been packaged in a medical coupling agent. The coupling model of the microstructure has been established as shown in Figure 5, and the material properties used in the simulation are shown in Table 2. In Figure 5, the red part represents the microstructure and the blue part represents the medical coupling agent, which are used to simulate the real working environment of the heart sound sensor. Coupling modal analysis results are shown in Figure 6. From Figure 6 it can be obtained that the resonant frequency of the microstructure is 975 Hz. The clinically-valuable heart sound frequency range is often concentrated in the range 20~600 Hz, so the microstructure can meet the requirements of the application.

**Figure 7:**
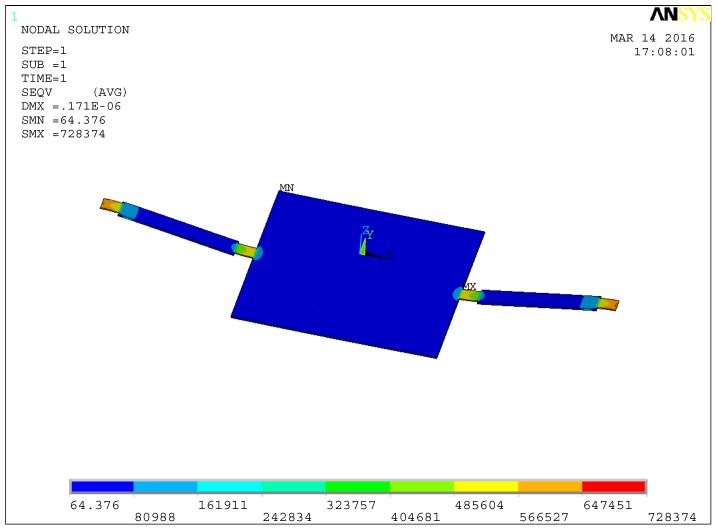
Stress distribution diagram.

**Figure 8:**
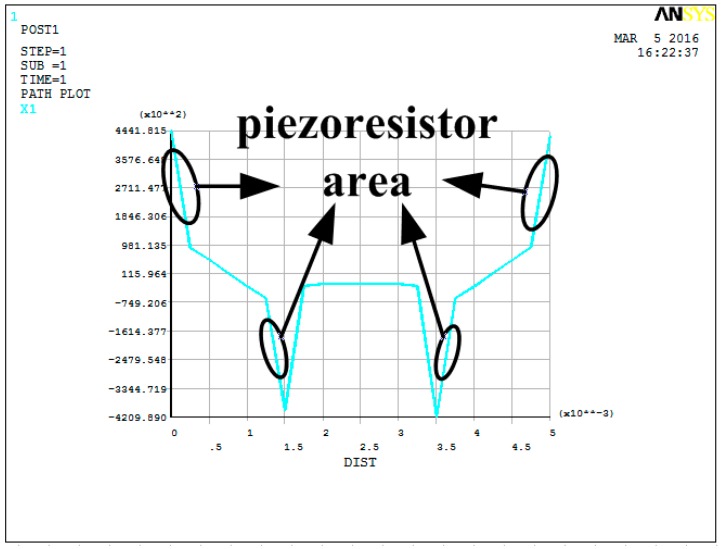
Stress distribution curve.

To verify the performances of stress concentration, the static simulation has been carried out. A 1 Pa load has been applied to the microstructure along the Z direction, and both ends of the beam have been fully constrained. The stress distribution diagram is shown in Figure 7. By defining the path, the stress distribution curve of the beam in the X direction has been extracted, as shown in Figure 8. The unit of the vertical axis in Figure 8 is Pa. The horizontal axis in Figure 8 corresponds to the whole length of the double-beam-block sensitive microstructure.

From the results of the static simulation, it can be seen that the stress is symmetrical on the beam. The maximum stresses occur in the stress concentration areas and have been greatly improved, so the piezoresistors should be arranged in these areas. Thus, it is verified that the detection sensitivity of the microstructure could be improved by stress concentration.

## 3. Fabrication

**Figure 9:**
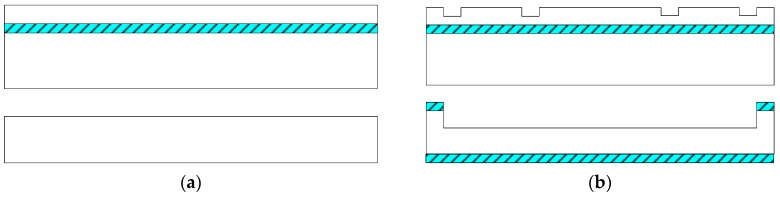

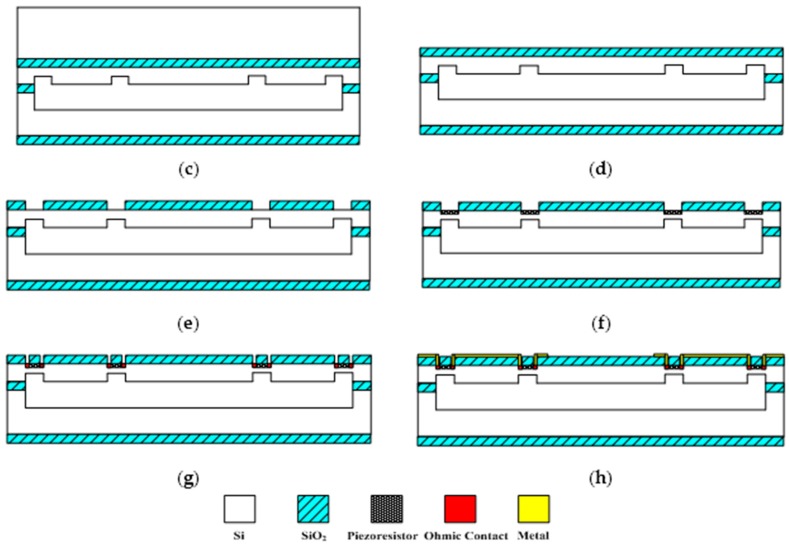
Process flow diagram: (**a**) preparing wafers; (**b**) RIE etching; (**c**) bonding; (**d**) TMAH etching; (**e**) etching; (**f**) implanting boron; (**g**) implanting denser boron; (**h**) sputtering and releasing.

**Figure 10:**
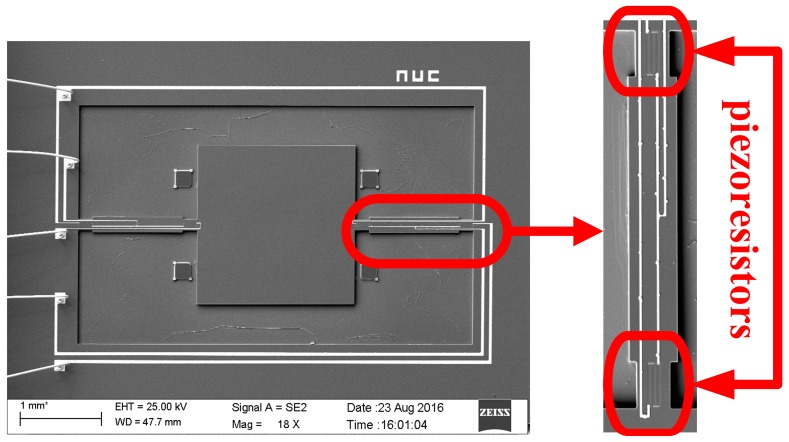
MEMS microstructure.

The sensitive microstructure of the MEMS electronic heart sound sensor is processed by MEMS technology based on a Si wafer and SOI (Silicon-On-Insulator) wafer. The fabrication procedures are illustrated in Figure 9a–h [18,19]: Figure 9a preparing the Si wafer and SOI wafer, a SOI wafer with a 20 µm device layer, 2 µm silicon dioxide layer and 400 µm substrate layer; Figure 9b RIE etching the device layer of the SOI wafer to form the back structure of the stress-concentrating grooves; oxidizing and RIE etching the Si wafer to form a cavity; Figure 9c bonding the device layer of the SOI wafer and Si wafer under nitrogen at 900~1100 °C; Figure 9d removing the substrate layer and silicon dioxide layer of the SOI wafer by TMAH (Tetramethylammonium Hydroxide) solution; Figure 9e RIE etching to form the stress concentration grooves; Figure 9f oxidation to form a 1000 Å silicon dioxide layer at 950 °C; etching and implanting boron with 100 KeV energy and 4 × 10^18^ cm^−3^ density to form the 5000 Ω piezoresistors at the stress concentration grooves; Figure 9g chemical vapor depositing to form a 1100 Å oxide layer on the top and ICP (Inductively Coupled Plasma) etching, implanting denser boron with 100 KeV energy and 4 × 10^21^ cm^−3^ density to form P+ area; Figure 9h sputtering to form metal interconnects, annealing to form ohmic contact under nitrogen at 1000 °C; ICP etching and releasing the structure. The MEMS microstructure is shown in Figure 10.

## 4. Package and Test

**Figure 11:**
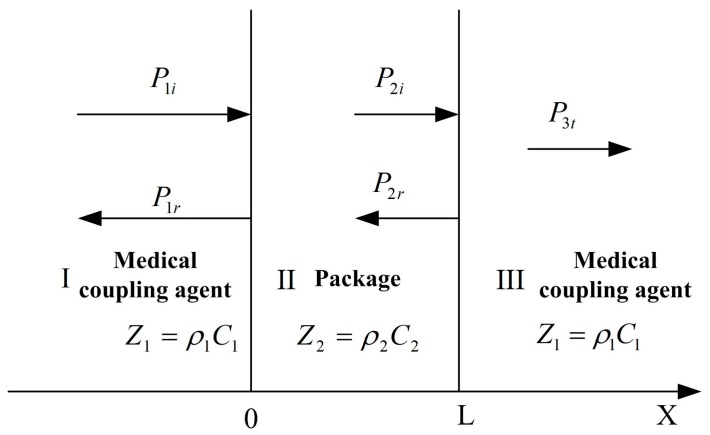
Theoretical model of the three-layer medium.

After the microstructure has been fabricated, it needs to be packaged to prevent the microstructure from being destroyed. At the same time, it should be ensured that the external acoustic signal can be maximally transmitted to the microstructure. Thus, acoustic impedance of the packaging structure should match with that of medical coupling agent to reduce the acoustic energy losses and improve the sensitivity of the MEMS electronic heart sound sensor. A theoretical model of the three-layer medium has been established to predict the sound-transparent performance of the packaging structure. Assuming that the acoustic wave is the plane wave when it propagates in the three-layer medium, the model is shown in Figure 11.

According to the above model, the sound-transparent coefficient can be expressed as Equation (3) [20]:

(3) $$T=\frac{1}{\cos^{2}(k_{2}L)+\frac{1}{4}{\left(\frac{Z_{2}}{Z_{1}}+\frac{Z_{1}}{Z_{2}}\right)}^{2}\sin^{2}(k_{2}L)}$$

where, *Z*_1_ and *Z*_2_, respectively, represent the characteristic impedance of the medical coupling agent and packaging structure, *k_2_* indicates the wave number, and *L* represents the thickness of the package. From Equation (3) it can be seen that when *Z*_1_ ≈ *Z*_2_, the sound-transparent coefficient *T* ≈ 1. In addition, it can be seen from Equation (3) that if the thickness of the sound-transparent package *L* is far smaller than the wavelength, that is *k*_2_*L*→0, *T* ≈ 1.

**Figure 12:**
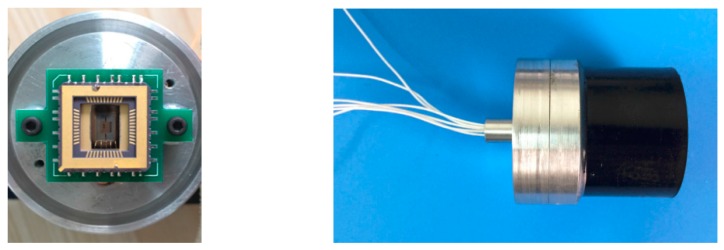
Prototype of the MEMS electronic heart sound sensor.

However, the inherent mechanical properties of the sound-transparent cap acting on the MEMS microstructure would influence the detection of heart sound signal, and the thinner the sound-transparent cap is, the lower its resonant frequency. Considering the sound-transparent performance and the resonant frequency, a polyurethane cap with 2 mm thickness has been selected and the preliminary packaged prototype of the MEMS electronic heart sound sensor is shown in Figure 12.

**Figure 13:**
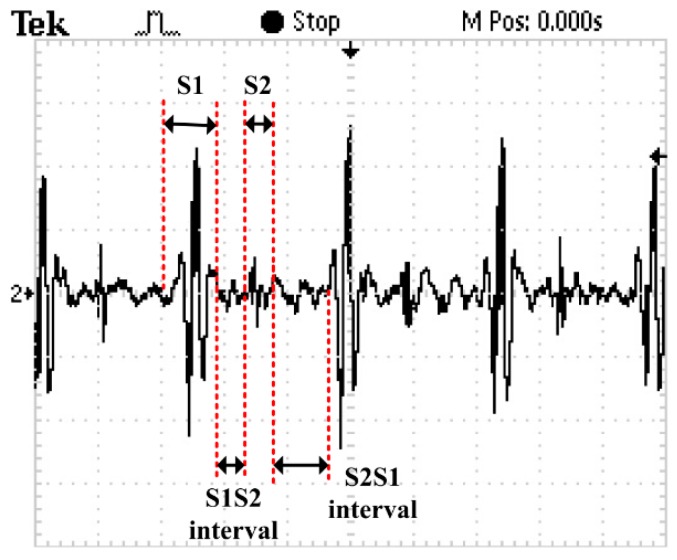
Waveform tested by the MEMS electronic heart sound sensor.

**Figure 14:**
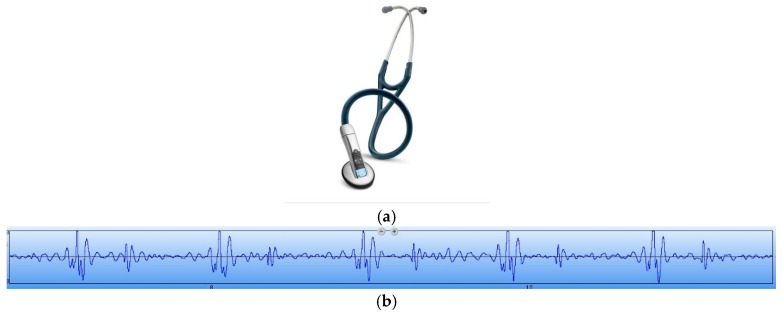
A 3200-type electronic stethoscope and its test results. (**a**) A 3200-type electronic stethoscope; and (**b**) the waveform tested by the 3200-type electronic stethoscope.

A random healthy young man has been selected as the auscultation object at room temperature. The MEMS electronic heart sound sensor has been connected to the Agilent 54622A oscilloscope (Palo Alto, CA, USA), and the waveform tested by it is shown in Figure 13. In order to verify the correctness of the test results, the results have been compared with that tested by a 3200-type electronic stethoscope from 3M (Figure 14). The 3200-type electronic stethoscope is a mature electronic stethoscope on the market, by which heart sound signals can be magnified 24 times and transferred to a computer for subsequent analysis by Bluetooth technology. It can be seen that the trend of the heart sound waveform signal tested by the MEMS electronic heart sound sensor is the same as that tested by the 3200-type electronic stethoscope. From the waveform it can be seen that the first and second heart sounds (S1 and S2) are obvious and his cardiac cycle is about 750 ms, and the heart rate is about 85 beats/min. The ratio of the S1S2 interval and S2S1 interval is approximately 1:2, which is consistent with the normal heart sounds standard.

**Figure 15:**
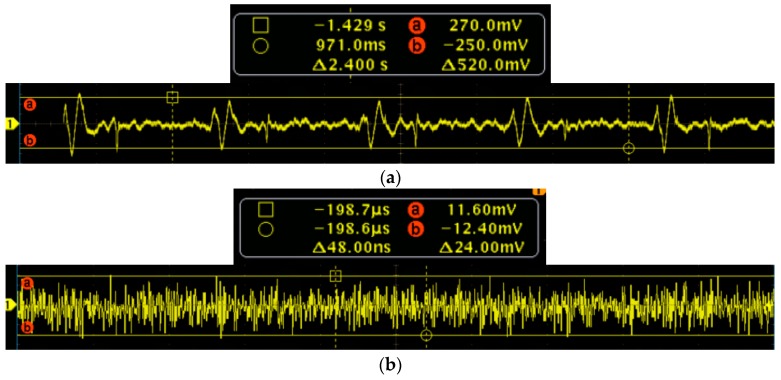
SNR test of the MEMS sensor. (**a**) Heart sound signals; and (**b**) output without the heart sound signals.

**Figure 16:**
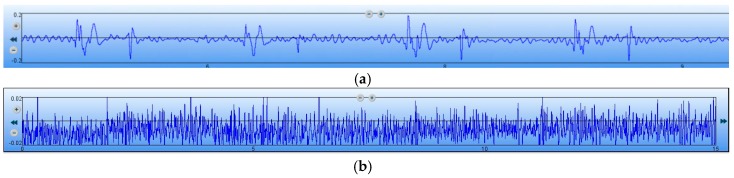
SNR test of the 3200-type electronic stethoscope. (**a**) Heart sound signals; and (**b**) output without the heart sound signals.

To further verify the performance of the MEMS sensor, the signal-to-noise ratio (SNR) testing experiments of the MEMS sensor and 3200-type electronic stethoscope have been carried out. Firstly, the MEMS sensor has been tested. The test results of the heart sound are shown in Figure 15a, and it is determined that the maximum of the heart sound signals is 270 mv. The output of the MEMS sensor without the heart sound signals are shown in Figure 15b, and it is determined that the noise signals is 12.4 mv. Therefore, by calculation, the SNR of the MEMS sensor is 27 dB. Then, the 3200-type electronic stethoscope has been tested. The test results of the heart sound are shown in Figure 16a, and it is determined that the maximum of the heart sound signals is 0.2. The output of the 3200-type electronic stethoscope without heart sound signals are shown in Figure 16b, and is determined that the noise signal is 0.02. Therefore, the SNR of the 3200-type electronic stethoscope is 20 dB. All of the above tests show that the SNR of the MEMS sensor is 7 dB higher than the 3200-type electronic stethoscope.

## 5. Conclusions

This study reveals a new attempt to implement MEMS technology and acoustic sensor technology in the traditional auscultation field of medicine. Heart sound vibration signals are transformed into electrical signals by the MEMS heart sound sensor to be displayed on an oscilloscope, by which the change from the traditional auscultation to heart sound has been realized, and the objectivity and accuracy of diagnosis has been improved. Due to the nature of MEMS technology, the MEMS electronic heart sound sensor has the advantages of small size, low power, visualization, ease of use, low cost, and good prospects for clinical application.

## Figures and Tables

**Table 1:** Parameters of the MEMS electronic heart sound sensor micro-structure.

Parameters (m)	Beam	Mass Block	Piezoresistor Area
Length	1.5 × 10^−3^	2.0 × 10^−3^	1.8 × 10^−4^
Width	2.0 × 10^−4^	2.0 × 10^−3^	1.4 × 10^−4^
Thickness	2.0 × 10^−5^	2.0 × 10^−5^	1.0 × 10^−5^

**Table 2:** Material properties.

Material	Silicon	Medical Coupling Agent	Polyurethane
Density (Kg/m^3^)	2330	1016	1070
Young’s modulus (10^11^ N/m^2^)	1.9	-	0.13
Poisson’s ratio	0.278	-	0.42
Sound velocity (m/s)	-	1520~1620	1520
Acoustic characteristic impedance (Pa·s/m)	-	1.5 × 10^6^~1.7 × 10^6^	1.5 × 10^6^
Slope of sound attenuation coefficient (dB/cm·MHz)	-	≤0.05	—
Viscosity (Pa·s)	-	≥15	—
pH	-	5.5–8.0	—
